# Impact of environmental enrichment and allostatic load induced by repeated mixing on the transcriptome of three porcine brain tissues

**DOI:** 10.1186/s13041-026-01351-w

**Published:** 2026-09-15

**Authors:** Liyan Deng, Marta Gòdia, J. Elizabeth Bolhuis, Martien A.M. Groenen, Barbara Harlizius, Martijn F.L. Derks, Lisette E. van der Zande, Severine P. Parois, Lu Luo, Zhonglin Tang, Ole Madsen

**Affiliations:** 1 Animal Breeding and Genomics Group, Wageningen University & Research, Wageningen, The Netherlands; 2 State Key Laboratory of Genome and Multi-omics Technologies, Shenzhen Branch, Guangdong Laboratory for Lingnan Modern Agriculture, Key Laboratory of Livestock and Poultry Multi-omics of the Ministry of Agriculture and Rural Affairs, Agricultural Genomics Institute at Shenzhen, Chinese Academy of Agricultural Sciences, Shenzhen, China; 3 Shenzhen Branch, Guangdong Laboratory of Lingnan Modern Agriculture, Key Laboratory of Livestock and Poultry Multi-omics of MARA, Agricultural Genomics Institute at Shenzhen, Chinese Academy of Agricultural Sciences, Shenzhen, China; 4 Adaptation Physiology Group, Wageningen University & Research, Wageningen, The Netherlands; 5 Topigs Norsvin Research Center, ’s-Hertogenbosch, the Netherlands; 6 Anses, French Agency for Food, Environmental and Occupational Health & Safety, Epidemiology Health and Welfare Unit, Ploufragan, France; 7 College of Animal Science & Technology, Nanjing Agricultural University, Nanjing, China

**Keywords:** Environmental enrichment, Allostatic load, Transcriptome, Hippocampus, Prefrontal cortex, Hypothalamus

## Abstract

Environmental enrichment and mixing represent two management procedures in pig production with opposing effects: while environmental enrichment can promote welfare by reducing stress, repeated mixing often induces stress and can negatively impact welfare. Despite their importance in pig husbandry, how these opposing management procedures affect brain function at the molecular level remains poorly understood. This study investigated how environmental enrichment and allostatic load induced by repeated mixing influence gene expression patterns across three porcine brain regions: hypothalamus, prefrontal cortex, and hippocampus. Using RNA sequencing, we analyzed brain tissues from 48 gilts subjected to a 2 × 2 factorial design comparing enrichment conditions (enriched vs. barren) and allostatic load (repeated vs. minimal mixing). While environmental enrichment showed minimal impact on gene expression, allostatic load resulted in 38 differentially expressed genes in the hippocampus, primarily involved in hormone regulation, protein transport, and neuronal activity. Weighted Gene Co-expression Network Analysis (WGCNA) identified modules moderately correlated with experimental conditions: six modules in hippocampus associated with allostatic load, and four modules in hypothalamus associated with enrichment. Functional annotation revealed that these modules were primarily enriched in biological processes related to synaptic signaling, neuron development, and metabolic activity. Hub genes from these modules were identified, and turned out not to overlap with differentially expressed genes. Our findings suggest that based on this experimental design, the hippocampus may be particularly responsive to stress-induced transcriptomic changes, while the molecular effects of the environmental enrichment appear more subtle. Overall, this study reveals limited but region-specific transcriptional responses to allostatic load in the porcine brain.

**Supplementary Information:**

The online version contains supplementary material available at https://doi.org/10.1186/s13041-026-01351-w.

## Introduction

Pigs are a key livestock species in food production systems. Housing and management conditions are important aspects of pig production, with numerous environmental factors that can cause stress in pigs [1, 2]. Sustained, cumulative stress has been associated with potential negative outcomes such as reduced feed intake, altered behavioral responses, and impaired immune functions [3–5]. These changes can ultimately affect the overall welfare of the animals.

Environmental enrichment, defined as a modification of the environment that improves the biological functioning of captive animals [6], plays a crucial role in promoting animal welfare. Studies have shown that an enriched environment can reduce stress and positively affect behaviors [7, 8]. For example, pigs raised in an enriched environment exhibit less damaging behaviors, such as tail or ear biting [9, 10]. Moreover, an enriched environment has been linked to improvements in cognitive skills and memory in rat studies [11, 12]. In line with this, environmental enrichment in pigs has been shown to enhance operant task and spatial learning [13] and working memory in a spatial task [14].

In contrast to environmental enrichment, repeated mixing, a common practice in pig farming used to manage group sizes and balance weight distributions [15], may have negative impacts on pigs. It may lead to a high allostatic load, i.e., the burden of cumulative wear and tear on the body due to adapting to chronic or repeated adverse situations [16, 17]. Mixing unfamiliar pigs has been shown to result in fights to establish dominance relationships, which are accompanied by physiological changes such as increased heart rate and elevated cortisol levels [18]. This practice has also been associated with variations in feed efficiency and daily weight gain [19]. Hence, environmental enrichment and repeated mixing can influence pig behavior and performance in different ways.

The brain plays a pivotal role in regulating various biological processes, including neuroendocrine signaling, cognition, and behavior. It is also susceptible to environmental factors and stress. Some studies in human and other animal species revealed that prolonged exposure to stress, leading to high allostatic load, can potentially impair brain function [17, 20], resulting in changes in cognition, memory, and behavior [21, 22]. Studies in rats have shown that environmental enrichment can mitigate or even reverse the neurobiological effects of chronic stress, reducing stress-induced neurotransmitter release in the prefrontal cortex, and potentially reverse the negative effects of early life adversity on stress responses [23, 24]. In pigs, changes in environmental enrichment and repeated mixing can induce neurobiological changes [25, 26], which may therefore alter brain transcriptome profiles and potentially influence the animal’s behavioral responses. A deeper understanding of these transcriptomic changes can help in optimizing management strategies and enhancing livestock resilience and welfare.

Each brain region contributes differently to stress regulation and adaptation. The hypothalamus, a key component of the hypothalamic-pituitary-adrenal (HPA) axis, regulates neuroendocrine stress responses through corticotropin-releasing hormone secretion [5, 27]. The prefrontal cortex is involved in higher-order processes such as cognition and reasoning [28, 29]. A previous study in mice has shown that unpredictable chronic mild stress exposure can significantly alter gene expression patterns in the prefrontal cortex [30]. The hippocampus, rich in glucocorticoid receptors, is susceptible to stress-induced structural and functional changes mediated by fluctuating glucocorticoid levels [31]. It has been reported that enriched environment can increase the abundance of proteins related to protein synthesis, microtubule assembly, vesicle-mediated transport and energy metabolism in the hippocampus of pigs [32]. In humans, chronic psychological stress has been shown to modulate the expression of various genes in the hippocampus, including those involved in neuronal plasticity and stress response [33].

While previous studies in human and other animals have investigated the effects of stress on brain function, the effects on the transcriptomic response in multiple porcine brain regions, and under different environmental or stress conditions remain to be studied. Previous transcriptomic research in pigs have mostly focused on a single brain region. For instance, a study investigating the relationship between the brain transcriptome and feed efficiency examined only the cerebral cortex [34]. Furthermore, transcriptomic studies of livestock exposed to both environmental enrichment and allostatic load induced by repeated mixing are absent, despite the significant implications of these factors for livestock welfare and productivity.

This study explored how environmental enrichment and repeated mixing shape brain transcriptome profiles in pigs by analyzing gene expression changes across key brain regions. Using bulk RNA sequencing, we examined the hypothalamus, prefrontal cortex, and hippocampus to identify differentially expressed genes under different experimental conditions. We investigated potential interactive effects between environmental enrichment and repeated mixing on gene expression patterns. Additionally, we applied Weighted Gene Co-expression Network Analysis (WGCNA) to detect gene modules that are co-expressed across samples to correlate them with the environmental enrichment conditions and allostatic load induced by repeated mixing.

## Materials and methods

### Animals, environmental enrichment and mixing treatments

The animals, and all experimental procedures, were based on the study by Luo et al. [35]. Briefly, 48 gilts from a single batch were randomly selected from a total of 384 three-way cross animals ((Landrace*Large White)*Synthetic). After weaning at an average age of 5 weeks (body weight 8.9 ± 0.2 kg), the piglets were transported to the experimental facility and assigned to a 2 × 2 factorial design comparing enrichment conditions (enriched vs. barren), and mixing frequency (repeated mixing vs. minimal mixing, i.e., only once) to represent different allostatic load levels (high vs. low). Twelve pigs were assigned to each experimental condition, ensuring balanced group sizes across all conditions. To minimize potential confounding factors, non-experimental variables such as pen size, temperature, and feed were kept identical across all groups. Furthermore, the animals per pen were balanced for their age, litter origin, and body weight. Consequently, for all downstream analyses, individual animals were treated as independent biological replicates.

Pigs in enriched conditions were provided with sawdust bedding, a variety of toys, access to a playground for 20 min every three days for four times in total, and daily positive human contact (gently brushed for 1 min daily for each pig). In contrast, pigs in barren conditions were kept in stimulus-poor pens without bedding, including two toys only (see Luo et al. [35] for details). In both treatments, the pigs were housed in groups of four. For the mixing treatment, pigs in the repeated mixing group were regrouped with three new unfamiliar pen mates every four days (on days 0, 4, 8, and 12 after arrival) to create high allostatic load [36, 37], while pigs in the minimal mixing group only experienced mixing at arrival (day 0). The experiment lasted for three weeks. During this period, physiological and phenotypic measurements, such as immune globulin levels reflecting natural antibodies and body weight, were recorded [35], see Supplementary Table [Table S1] for a full overview.

### Sample collection, RNA extraction and sequencing

The 48 gilts were slaughtered at an average age of 9 weeks (body weight 20.5 ± 3.7 kg). Tissue samples from different parts of the brain were collected from each animal immediately after slaughter and preserved in RNAlater (Thermo Fisher Scientific) at −80 °C until further use. For the current study, tissues from the hypothalamus, prefrontal cortex and hippocampus were available. Hypothalamus tissues from 3 animals could not be collected due to technical issues.

Total RNA was extracted using RNeasy kit (Qiagen) according to the manufacturer’s guidelines. The extracted RNA went through quality control procedures, including quantification using NanoDrop 1000 (Thermo Fisher) and Qubit RNA BR Assay kit (Invitrogen). Extracted RNA averaged 76.0, 107.0 and 107.9 ng/µl for hypothalamus, prefrontal cortex, and hippocampus, respectively. RNA integrity was evaluated with the Bioanalyzer Agilent RNA 6000 Nano kit (Agilent Technologies). All samples presented RNA Integrity (RIN) values ≥ 9. RNA was poly(A) enriched using NEBNext^®^ Poly(A) mRNA Magnetic Isolation Module (New England Biolabs), followed by library preparation with the NEBNext^®^ Ultra™ Directional RNA Library Prep Kit (New England Biolabs). Sequencing was conducted on the Illumina NovaSeq 6000 platform, generating stranded 150 bp paired-end reads.

### Quality control and read mapping

Quality of raw RNA-seq reads was evaluated using FastQC 0.11.9 [38]. Low-quality bases and adaptor sequences were trimmed by Trim Galore 0.6.10 [39], using default settings. We aligned our filtered reads to the porcine reference genome Sscrofa11.1 [40] (Ensembl version 112) using STAR 2.7.11b with default parameters [41]. Reads that had an alignment MAPQ score smaller than 30 were removed by SAMtools 1.18 [42].

### Differential expression analysis and functional enrichment

Read counts were obtained using htseq 2.0.5 [43] with the parameter “--stranded = reverse”, followed by TMM normalization of count per million (CPM) using EdgeR 4.0.16 [44]. Principal Component Analyses (PCA) were performed with the R package DESeq2 [45] to evaluate the effects of environmental enrichment and allostatic load on overall gene expression. To visualize similarity of gene expression patterns among individuals, we generated heatmaps based on pairwise correlation and the Euclidean distance between samples using the ComplexHeatmap R package [46]. Volcano plots were generated using the EnhancedVolcano R package [47] to show genome-wide expression of genes across different tissues.

Differential expression analysis was conducted using DESeq2 [45]. To evaluate the main effects of environmental enrichment and allostatic load, we utilized the simple design formulas (~ condition and ~ allostatic_load) for these two experimental settings, respectively. Furthermore, we assessed the additive and interaction effects using a combined model (~ condition + allostatic_load) and a full model (~ condition + allostatic_load + condition: allostatic_load). We did not include covariates in any of our models, as the experimental design has controlled non-experimental factors, and the animals were already balanced across groups. Genes with an absolute foldchange |FC| ≥ 1.5 and a false discovery rate (FDR) < 0.05 were considered differentially expressed between different conditions. Functional enrichment including the Gene Ontology (GO) and Kyoto Encyclopedia of Genes and Genomes (KEGG) pathways of the differentially expressed genes (DEGs) was assessed using the ClusterProfiler R package [48], with all expressed genes in each tissue serving as the background gene set, respectively. FDR correction was applied to the p-values obtained.

### Gene co-expression analysis

WGCNA was performed using the WGCNA R package [49]. For this analysis, we kept genes with an average expression equal or greater than 1 CPM across all samples and applied a log_2_ (CPM + 1) transformation to the original expression of genes. Hierarchical clustering was used to detect and remove outlier samples. Next, the soft-threshold power (β) was calculated for each tissue to construct a scale-free topology network. A power of 14, 6, and 12 was chosen for the hypothalamus, prefrontal cortex, and hippocampus, respectively, with the criterion of R² > 0.8. Gene modules were identified using hierarchical clustering based on the topological overlap matrix (TOM). A minimum size of 30 genes was required and modules with similar expression profiles were merged using a height cutoff of 0.25.

Module eigengenes (MEs), the first principal component of each module, represent the weighted average of gene expression within the module. We performed Pearson correlations between gene modules and experimental conditions and phenotypes. Candidate modules were selected for downstream analysis based on their correlation levels and nominal p-values, using the criteria of |R| > 0.3 and *p* < 0.05. The gene significance and module membership were calculated to evaluate the importance of genes in their respective modules. We investigated genes in the key modules with functional enrichment analysis, including the GO and KEGG analysis, using the ClusterProfiler R package [48] with expressed genes as background. FDR correction was applied to the p-values obtained.

Intramodule connectivity was calculated to assess the correlation between each gene and all other genes within the same module. We selected the top 30 genes with the highest intramodule connectivity in a module as hub genes. We then imported the networks consisted of hub genes into Cytoscape for visualization [50].

The hub genes and DEGs were further examined through PigGTEx [51], animalTFDB [52], GeneCards [53], and existing publications to elucidate their roles in biological processes.

## Results

### Transcriptome profiling and differential analysis reveal transcriptional alterations in the hippocampus and hypothalamus under allostatic load conditions

**Fig. 1:**
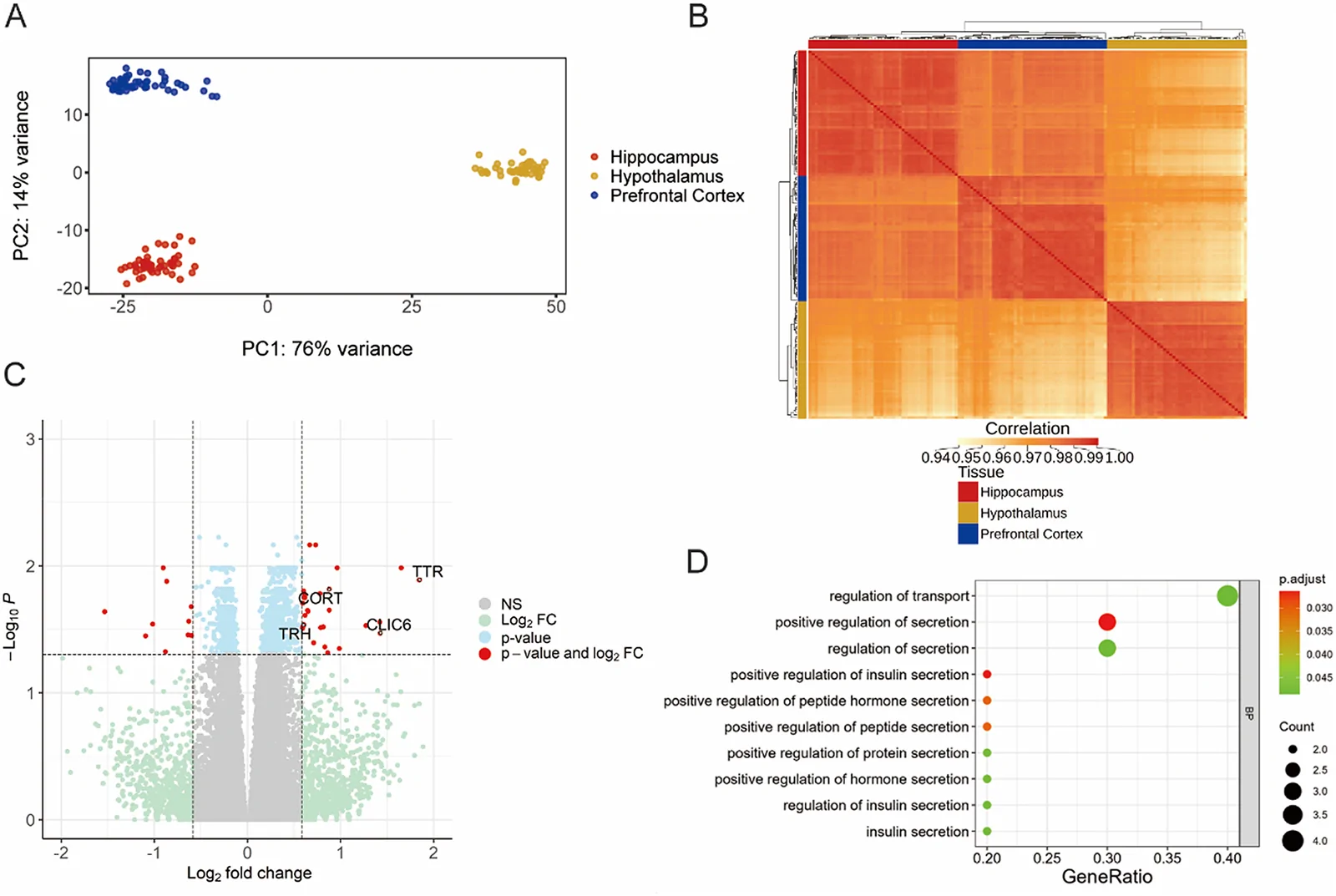
Gene expression patterns and differential gene expression analysis across brain regions. **A**) Principal Component Analysis (PCA) of gene expression data among prefrontal cortex, hippocampus, and hypothalamus samples. **B**) Correlation heatmap displaying pairwise sample correlations based on gene expression profiles. **C**) Volcano plot showing the differentially expressed genes (DEGs) in hippocampus with significance thresholds. Several significant stress-related DEGs (*CORT*, *CLIC6*, *TTR*, and *TRH*) are highlighted in the plot. **D**) Enriched biological processes for the DEGs in hippocampus. NS: Not Significant; FC: Fold Change

The RNA-seq generated an average of 23 million paired-end reads per sample across all three tissues. On average, 96% of the reads mapped to the porcine reference genome assembly Sscrofa11.1 (annotation version 112). A total of 15,470, 15,368, and 15,425 genes were found expressed at CPM ≥ 1 in hypothalamus, prefrontal cortex and hippocampus, respectively. PCA analysis revealed distinct clustering of the three tissues [Figure 1A]. The hypothalamus was clearly separated from the prefrontal cortex and hippocampus along PC1, while the hippocampus and prefrontal cortex were separated along PC2. Sample correlation showed high similarity across all samples, with strongest correlations observed within samples from the same tissue type [Figure 1B]. We therefore included all samples for the different differential expression analyses. We also examined the effects of enrichment and allostatic load levels on gene expression, but the clustering among individuals showed no clear distinction [Fig. S1-S3].

To further investigate transcriptional differences, we performed differential expression analyses for both enrichment and allostatic load conditions separately. The analyses of different enrichment conditions (enriched vs. barren) did not result in any DEGs for any of the three tissue types studied, indicating that enrichment had no significant impact on gene expression. For the analysis of different allostatic load levels (repeated vs. minimal mixing), we identified 38 DEGs in the hippocampus [Figure 1C], 1 DEG (*MPZ*) in the hypothalamus, and 0 DEGs for the prefrontal cortex (Table S2). No significant interaction effect was found between enrichment conditions and allostatic load, suggesting these two experimental conditions influence gene expression independently.

**Fig. 2:**
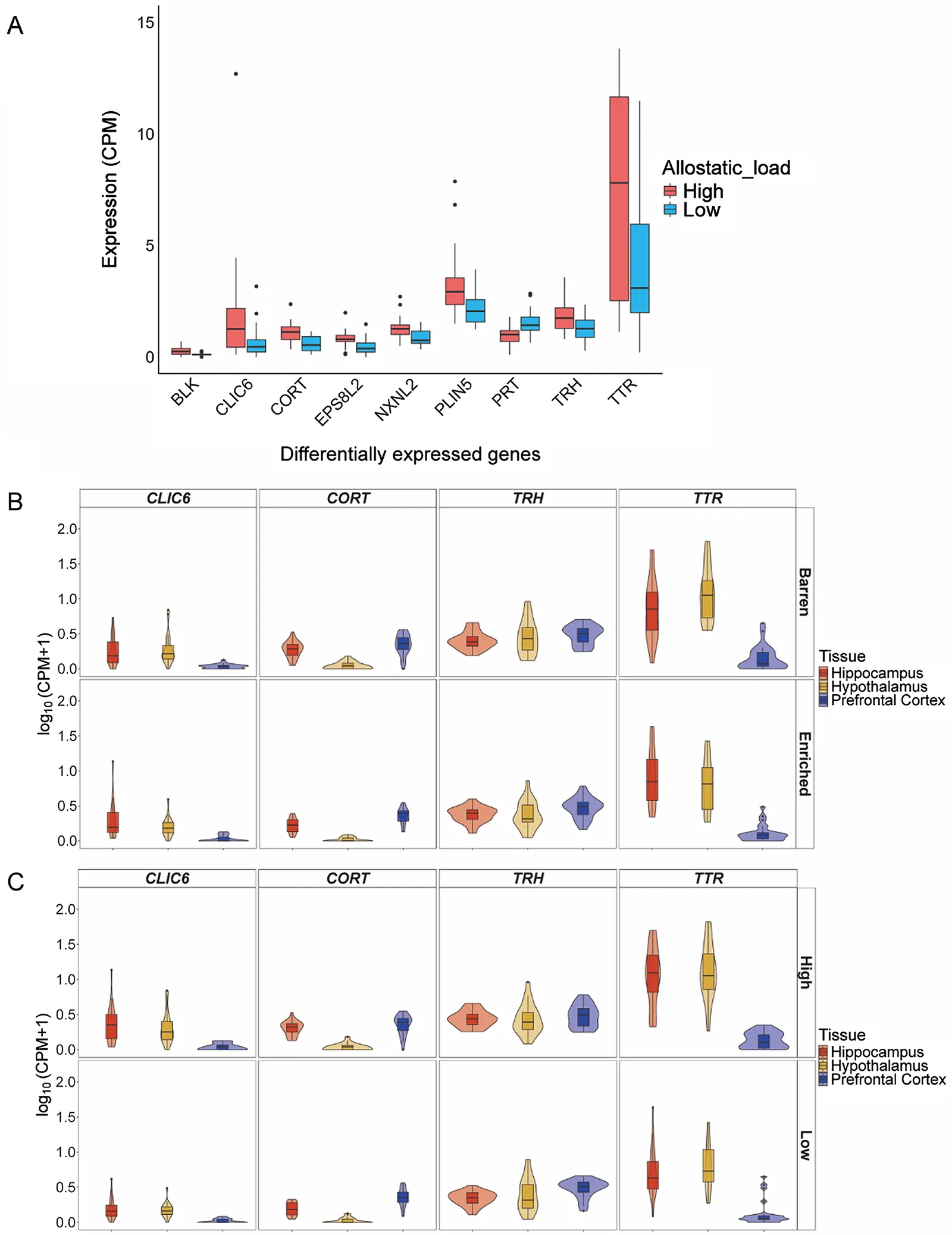
Expression patterns of selected DEGs in hippocampus and stress-related DEGs across different brain regions. **A**) Expression levels of some of the DEGs found in hippocampus for high versus low allostatic load (e.g. repeated vs. minimal mixing). **B**) Expression pattern of four stress-related DEGs (*CORT*, *CLIC6*, *TTR*, and *TRH*) under different enrichment conditions (enriched vs. barren) across all three brain regions. The expression values are log-transformed (log_10_[CPM + 1]) for better visualization. **C**) The expression pattern of the same genes under different allostatic load conditions

GO enrichment analyses of the DEGs in the hippocampus revealed that these genes were significantly associated with processes related to regulation of protein and hormone transport as well as hormone, peptide and insulin secretion [Figure 1D]. KEGG analysis did not find any significant pathway. Further analysis of the DEGs from the hippocampus indicated that most were related to neuronal activity, hormone regulation during stress, immune response, and metabolism. We selected some DEGs for their relevance with stress response and other significant biological processes based on previous literature. Notably, many of these DEGs were upregulated in the hippocampus under high allostatic load (repeated mixing) compared to low allostatic load (minimal mixing) [Figure 2A]. Among these DEGs, *TTR*, which encodes transthyretin, showed the greatest difference in expression between high and low allostatic load condition. The only gene downregulated under high allostatic load condition was *PRTG*, which is associated with neurogenesis [54]. These genes exhibited low to moderate gene expression profiles in hypothalamus, then, their expression in this, as well as the other tissues, was extracted for further examination. For example, for four stress-related genes (*CORT*, *CLIC6*, *TTR*, and *TRH*), we examined expression patterns across the prefrontal cortex, hippocampus, and hypothalamus under different enrichment conditions and allostatic load levels. We observed variation of gene expression of the selected genes between tissue types. *TTR* and *CLIC6* exhibited higher expression in both hippocampus and hypothalamus compared to the prefrontal cortex across all conditions. In contrast, *CORT* showed minimal expression in hypothalamus but higher levels in hippocampus and prefrontal cortex. [Figure 2B and C].

### Weighted gene co-expression network analysis uncovers functional modules and hub genes linked to neuronal activity

**Fig. 3:**
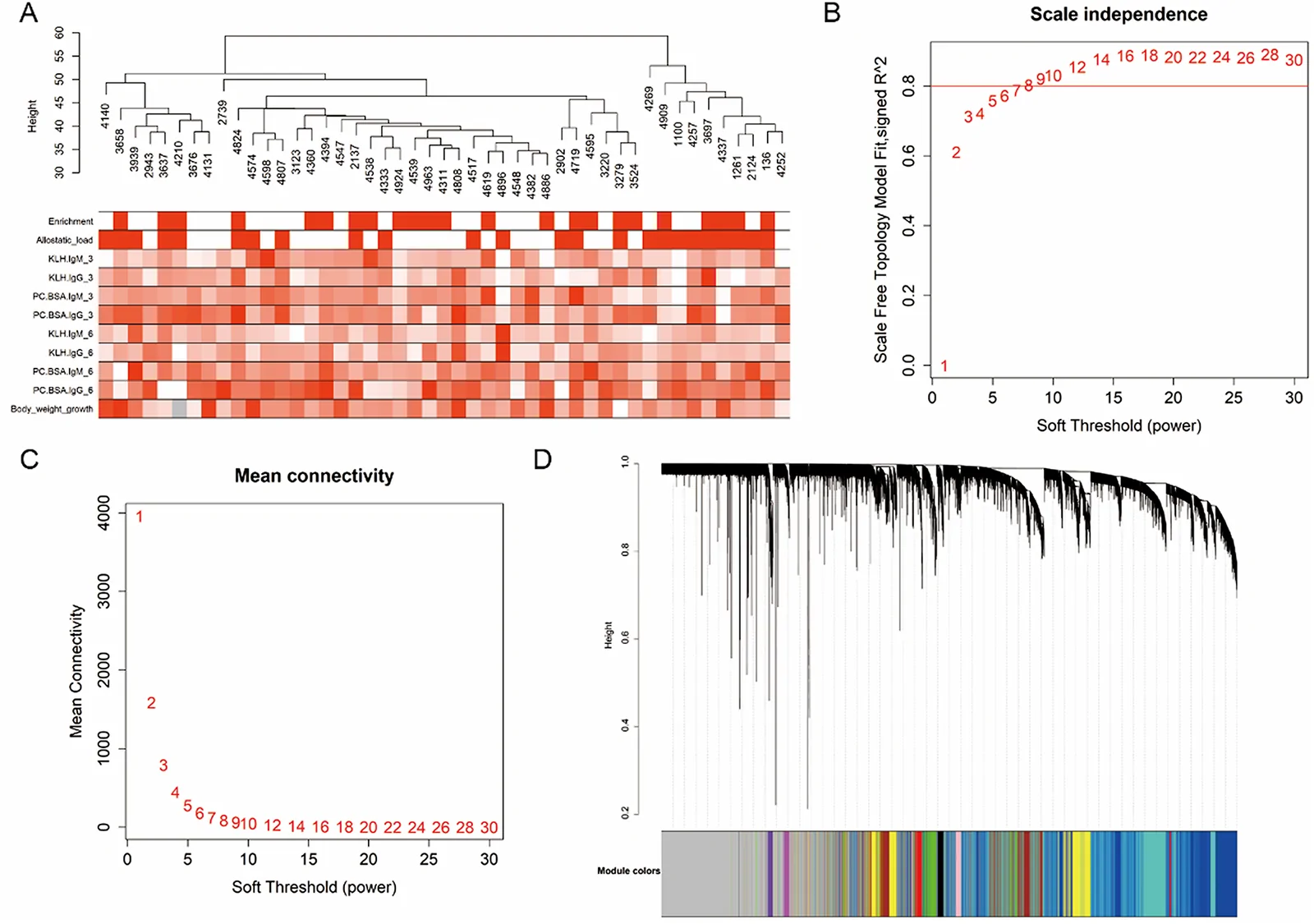
WGCNA and modules identified in the hippocampus. Similar analyses were performed for hypothalamus and prefrontal cortex, with results shown in Supplementary Figures [Fig. S4-S5]. **A**) Hierarchical clustering dendrogram (top) groups samples based on gene expression similarity. The heatmap (bottom) shows the relationship between samples and phenotypic measurements. For categorical variables (“Enrichment” and “Allostatic_load”), red indicates samples that strongly associate with one condition (e.g., environmental enrichment or high allostatic load), while white indicates association with the alternative condition. For continuous variables, red intensity represents the strength of positive correlation between the sample’s gene expression pattern and the phenotypic measurement. Detailed phenotypic measurements are provided in Table S1. **B**) Analysis of scale independence to determine optimal soft-thresholding power for network construction. **C**) Mean connectivity plot showing network connectivity as a function of soft-thresholding power. **D**) Gene dendrogram of hippocampus constructed based on the measurement of dissimilarity. The color band indicated the clustering of genes assigned into distinct co-expression modules. Acronyms of phenotypes are provided in Table S1

Gene modules were identified using WGCNA, ensuring that genes within each module exhibited both high co-expression levels and topological overlap similarity. This approach allows us to uncover co-regulated gene networks that may be functionally relevant to environmental enrichment or allostatic load conditions. Pearson correlation was applied to cluster the samples and to remove outliers [Figure 3A]. We selected a soft-threshold power of 14, 6, and 12 (R² > 0.8) for the hypothalamus, prefrontal cortex and hippocampus, respectively to construct the network. Subsequently, the adjacency matrix was converted to TOM based on the soft-threshold of selection [Fig. 3B and C]. We used the unsigned TOM to capture both positive and negative correlations between genes. Through average hierarchical clustering and dynamic tree clipping a total of 15, 12 and 12 gene modules were identified for the hypothalamus, prefrontal cortex and hippocampus, respectively [Figure 3D].

**Fig. 4:**
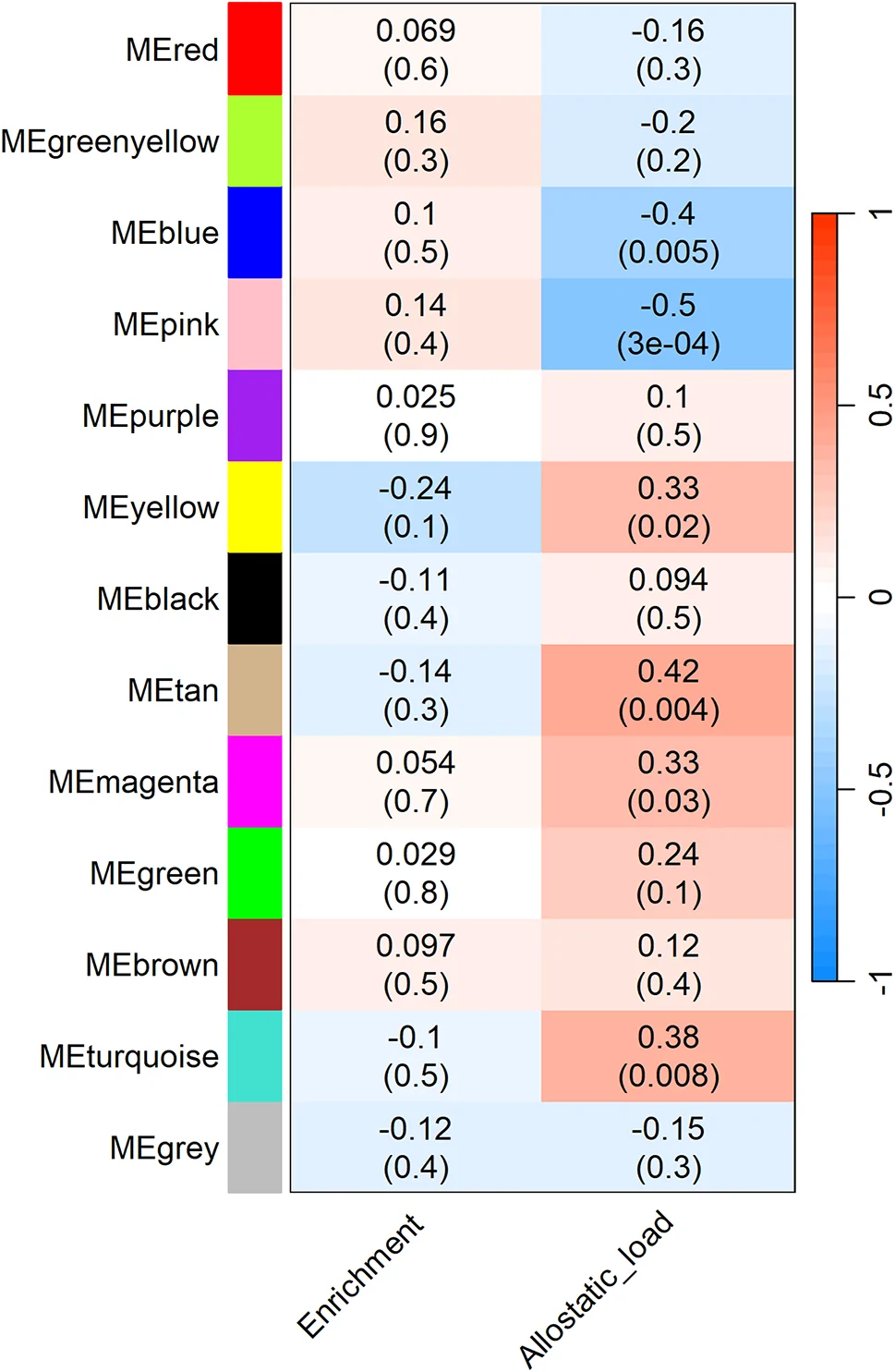
Module-trait relationships in hippocampus. Correlation heatmap showing module-trait relationships between identified gene modules (rows) and experimental conditions (columns), with red indicating positive correlation and blue indicating negative correlation. The values in each cell represent the Pearson correlation coefficient (R), with the corresponding nominal p-value in parentheses. Module eigengenes (MEs) represent the first principal component of gene expression within each module and serve as a summary profile of the overall expression pattern for genes in that module. Module-trait relationships for other phenotypes are in Fig. S6-S8

**Fig. 5:**
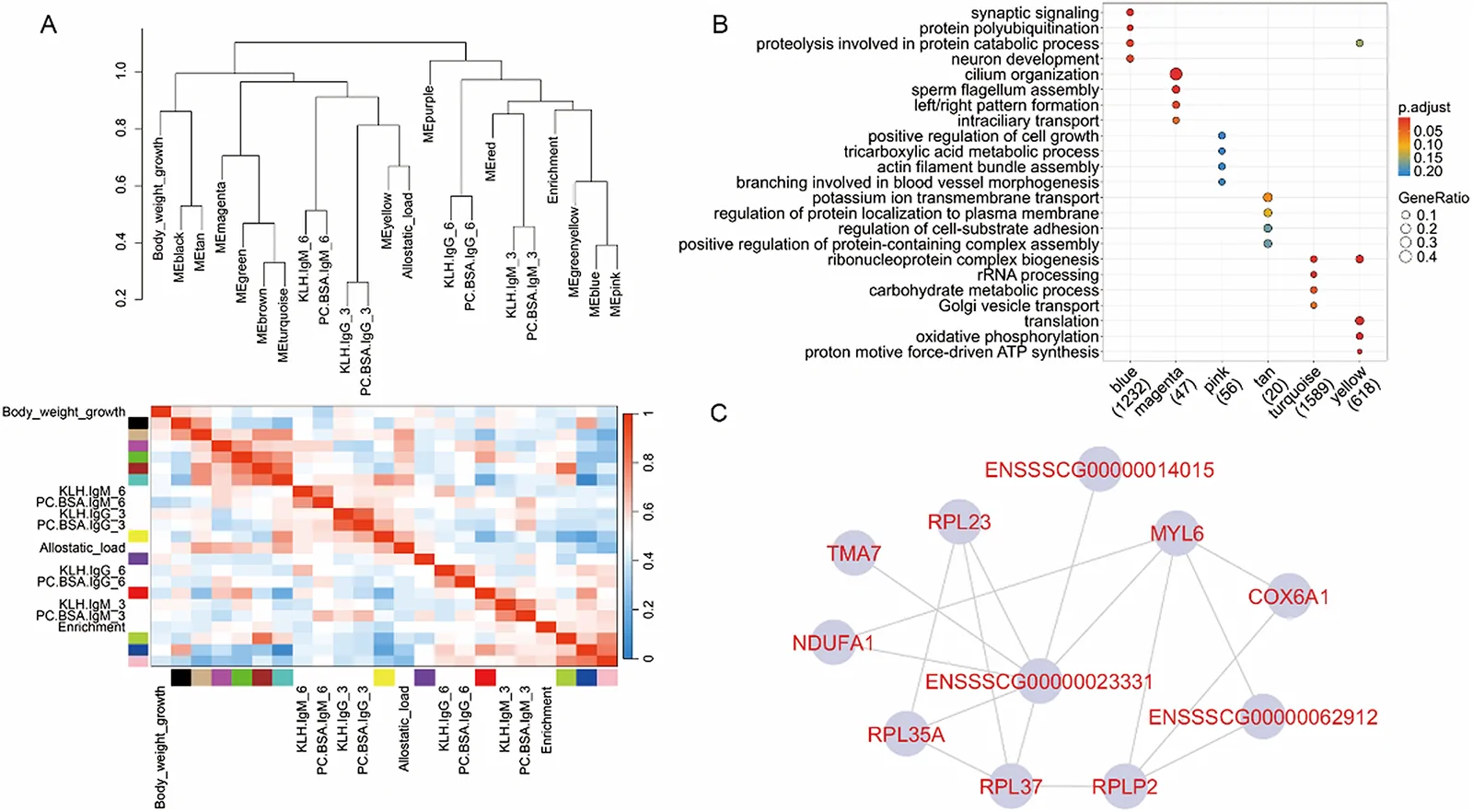
Gene co-expression network in the hippocampus and its functional implications. **A**) Hierarchical clustering dendrogram of module eigengenes (top) and their correlation heatmap (bottom). **B**) GO enrichment analysis of significant modules showing key biological processes. **C**) Network visualization of hub genes and their interactions within the yellow module

Correlation analyses between module eigengenes and phenotypic measurements revealed multiple modules correlated (|R| > 0.3, *p* < 0.05) with these measurements across all brain regions. In the hippocampus, six modules (blue, pink, yellow, tan, magenta, and turquoise) correlated with allostatic load [Figure 4, Fig S6], while in the hypothalamus, four modules (brown, salmon, magenta, and turquoise) associated with enrichment [Fig. S7]. In the prefrontal cortex, the purple module correlated with allostatic load and the red module with environmental enrichment. Notably, the purple module in the prefrontal cortex was also the only one showing modest correlation (*R* = 0.33, *p* = 0.02) with growth during the experiment [Fig. S8]. While some modules correlated with immune globulin levels (such as anti-PC-BSA IgG measured at week 3), these associations were sparse and inconsistent across modules or brain regions [Fig. S8]. In addition, we present a dendrogram and its accompanying heatmap of module eigengenes in the hippocampus to further illustrate the correlation patterns between module eigengenes and phenotypic measurements [Figure 5A]. Notably, enrichment and allostatic load conditions were in separate clades of the dendrogram, and showed little correlation in the heatmap. This could indicate that the two treatments affect gene expression and molecular pathways independently. Some gene modules share moderate correlations, suggesting potential functional relationships or co-regulation among these gene networks.

To understand the biological relevance of these modules, we compared GO terms enriched in these modules. For clarity, we present detailed results from the hippocampus, since it exhibited most of the DEGs and several modules correlated with the allostatic load condition. The result showed distinct molecular and cellular functions across these modules [Figure 5B]. We calculated top 30 hub genes based on intramodular connectivity, and the functions of these genes from each module essentially supported the results from the enrichment analysis. For instance, the hippocampus yellow module’s hub genes included ribosomal proteins (*RPLP2*, *RPL23*, *RPL37*) and mitochondrial related genes (*COX6A1*, *NDUFA1*) which may contribute to its enriched biological processes in protein biosynthesis and energy production [55–57] [Figure 5C]. While hub gene analysis was biologically informative for most modules, it was not feasible for some modules with limited gene numbers, such as the hippocampus magenta module. Notably, there was no overlap between the hub genes from these modules and the DEGs identified [Table S2].

## Discussion

Repeated mixing and environmental enrichment can influence pig behavior and physiological response, ultimately affecting welfare and performance. Luo et al. [35] reported some effects of enriched housing and repeated mixing on hormone, immune and growth-related measurements. In contrast with a previous experiment, in which more elaborate enrichment was provided from birth onwards [58], the enriched housing used in the current study did not increase resilience, i.e. the speed of recovery, after a sickness and frustration challenge. But it appeared to reduce stress-related and damaging behaviors, and mildly affected physiological responses [35]. Pigs housed in the enriched environment exhibited less aggressive behavior during mixing than those in barren housing. Surprisingly, repeated mixing did not exacerbate stress in barren-housed pigs but seemed to mitigate some of its negative effects. Growth was unaffected by enrichment but unexpectedly increased by repeated mixing conditions, raising questions about to what extent repeated mixing increases allostatic load. However, some natural antibodies appeared to be influenced by the interaction between enrichment and repeated mixing.

Given these results, we examined whether enrichment and allostatic load conditions led to distinct gene expression patterns in three brain tissues and whether phenotypic differences were consistent with gene expression patterns. PCA of gene expression data revealed clear separation among brain tissues, but no major differences among individuals subjected to enrichment or allostatic load conditions. This suggests that, in this experimental setup, these factors had a limited impact on the global gene expression within the three porcine brain tissues analyzed, at least not in PC1 and PC2, which together accounted for 90% of the variance.

Our differential expression analyses, which targeted per gene basis, also supported these findings. Only a small number of DEGs associated with allostatic load were observed. No DEGs were identified associated with the enrichment condition, indicating that the enrichment as applied in this study has minimal effect on transcriptomic changes in the three brain tissues. Based on the functional roles of these brain regions, we anticipated that the hypothalamus, as the primary regulator of the HPA axis, which plays a role in stress response, would present some transcriptomic response to allostatic load conditions. We also expected that the prefrontal cortex, involved in complex cognitive processes, would also exhibit some transcriptomic changes due to the experimental setup. However, our results did not conform to these expectations. In the hypothalamus, only one DEG was identified: *MPZ*, a gene specifically expressed in Schwann cells. This gene encodes a type I transmembrane glycoprotein, which is essential for myelination in the peripheral nervous system [59]. The lack of genes with altered expression profiles aligns with the small effects found in Luo et al.’s study, suggesting that the contrast in housing conditions may have been small [35]. In contrast, more DEGs were identified in the hippocampus under different allostatic load conditions. GO enrichment analysis indicated that these genes are involved in protein and hormone transport and secretion. While repeated mixing was designed to induce high allostatic load, however, the relatively low number of DEGs aligns with the limited physiological differences observed in Luo et al.‘s study [35].

We examined these DEGs and found several related to hormone regulation, neural activity, and general metabolic homeostasis. For instance, genes implicated in stress response and neuroendocrine regulation, such as *TTR*, *CORT*, and *TRH*, were altered. *TTR* has been reported to be elevated under chronic stress in the mouse hippocampus [60], and *CORT* is a gene highly expressed in prefrontal cortex and brain [51]. Both *CORT* and *TRH* play established roles in regulating the HPA and hypothalamic-pituitary-thyroid (HPT) axes during stress adaptation [61–64]. Other DEGs are associated with neural signaling (e.g., *CLIC6* [65, 66]), immune function (e.g., *BLK* [67, 68]), and lipid metabolism (e.g., *PLIN5* [69, 70]). The expression changes of these genes align with our observation that repeated mixing affected hippocampal gene expression only modestly. The hippocampus may be particularly sensitive to stressors, since glucocorticoid and mineralocorticoid receptors are highly expressed in hippocampus [71]. Given the limited transcriptomic alterations observed, the broader implications of these findings for pig husbandry practices require further investigation.

Our WGCNA analysis revealed distinct gene co-expression patterns associated with experimental conditions across brain regions. Six modules from the hippocampus correlated with mixing conditions, suggesting its sensitivity to stress-related changes. The hypothalamus, in contrast, appeared more responsive to enrichment, with four modules showing associations. The prefrontal cortex demonstrated a more limited response overall, with only two modules showing correlations: one to allostatic load (purple module) and one to enrichment (red module). While the purple module in prefrontal cortex also showed a modest correlation with body weight growth, we found no consistent associations between gene modules and physiological measurements (e.g. immunoglobulin levels and body weight) across brain regions. However, Luo et al. reported that natural antibody levels, including KLH-IgM and KLH-IgG, were significantly affected by the combination of enrichment and repeated mixing [35].

To further understand the biological relevance of these modules, we performed GO enrichment analyses. In the hippocampus, five modules (blue, magenta, tan, turquoise and yellow) showed significant enrichment. These modules were mainly related to synaptic signaling and neuron development, transmembrane transport, rRNA processing and energy dependent metabolic processes and protein synthesis. In the hypothalamus, three modules (brown, magenta, and turquoise) returned significant enrichment results, and these modules were associated with synaptic signaling and neuron development, energy-dependent metabolic processes, translation, and protein transport. Notably, the functional annotation of modules between the hippocampus and hypothalamus was highly similar. For instance, the blue module in the hippocampus shared similar molecular and cellular functions with the blue and magenta modules in the hypothalamus. This similarity of co-expressed gene modules across brain regions suggests shared biological processes, particularly those related to neural signaling and metabolic activity.

Identification of hub genes based on highest intramodule connectivity revealed overlapping genes between the hippocampus blue module and hypothalamus turquoise module, including *MYO5A*, *TTC3*, *DDX3X* and *MAP1B*. All these genes have been reported to be associated with neuronal development, synaptic signaling or brain functions [72–77], indicating the biological relevance of these co-expressed modules in regulating neuronal processes. However, none of the hub genes was found as DEGs, further suggesting the mild impact of our experimental conditions on gene expression.

Previous studies in mice and fish have shown that environmental enrichment can influence the brain transcriptome, primarily by modulating the expression of genes related to stress responses, metabolism, and neuroplasticity [78, 79]. However, in our study, we did not observe significant transcriptomic changes associated with environmental enrichment. This may be due to the type of enrichment used, such as the duration, frequency of the enrichment exposure, as well as the sample collection process or timing, which may eventually diminish the transcriptomic effects. The enriched and barren housing conditions may not have provided sufficient contrast to produce robust biological effects, as Luo et al. reported [35], weaker physiological responses were identified compared to other environmental enrichment studies in pigs, such as the study from Parois et al. [58]. Moreover, previous research has shown that allostatic load can alter the brain transcriptome. For instance, Lisowski et al. reported robust upregulation of the *TTR* gene and downregulation of genes involved in insulin secretion, neuropeptide hormone activity, and dopamine receptor mediated signaling (such as *CLIC6*) in the prefrontal cortex of mice exposed to chronic stress [80]. Notably, both *TTR* and *CLIC6* were among the DEGs identified in our study, but in the hippocampus rather than in the prefrontal cortex. We also observed DEGs and co-expression modules associated with stress, metabolism, and immune responses. While these observations align with previous studies, our results indicate that the transcriptomic changes are modest and mostly restricted to the hippocampus under the present experimental conditions. The broader biological implications of these findings for pig husbandry practices require further investigation.

## Conclusion

This study explored the effects of environmental enrichment and repeated mixing (intended to induce high allostatic load) on gene expression in three porcine brain regions using differential expression analysis and WGCNA. Allostatic load resulted in a limited number of DEGs in the hippocampus, primarily associated with pathways such as neuronal development, synaptic signaling, and metabolism. In contrast, the enrichment condition used in this study had minimal impact on overall gene expression in the three porcine brain tissues studied. WGCNA identified several gene modules in the hippocampus and hypothalamus that were correlated with allostatic load and enrichment, yet no associations were found with growth or immune-related phenotype measurements. Hub genes identified in these modules did not overlap with DEGs, suggesting that these experimental conditions may affect co-expression networks without altering gene expression levels. The brain is a highly complex organ, and our findings suggest that the transcriptional responses to environmental enrichment and repeated mixing are subtle. Consequently, the modest effects observed suggest that the biological impact of these conditions might involve more intricate mechanisms that cannot be fully captured by bulk gene expression analysis alone. To better understand the impact of allostatic load and environmental enrichment on brain function, further research with alternative experimental designs is still needed.

## Supplementary Information

Below is the link to the electronic supplementary material.

Supplementary Material 1

## Data Availability

The RNA-seq data generated in this study are available in the European Nucleotide Archive (ENA) repository under the project accession PRJEB105611.
